# Anaerobic fermentation of rice bran using rumen liquor for desirable chemical changes as animal feed

**DOI:** 10.5455/javar.2022.i642

**Published:** 2022-12-31

**Authors:** Khan Md. Shaiful Islam, Mabrouk Elsabagh, Renlong Lv, Hoang Lam Dang, Toshihisa Sugino, Taketo Obitsu

**Affiliations:** 1 Department of Animal Nutrition, Bangladesh Agricultural University, Mymensingh-2202, Bangladesh; 2 Department of Animal Production and Technology, Faculty of Agricultural Sciences and Technologies, Nigde University, Nigde 51240, Turkey; 3 Department of Nutrition and Clinical Nutrition, Faculty of Veterinary Medicine, Kafrelsheikh University, 33516 Kafr El-Sheikh, Egypt; 4 Tropical Crop Germplasm Research Institute, Chinese Academy of Tropical Agricultural Sciences, Haikou, 571101, Hainan, China; 5 Department of Agriculture, Forestry and Aquaculture Science, Hung Vuong University, Phu Tho, Vietnam; 6 Graduate School of Biosphere Science, Hiroshima University, Higashi-Hiroshima, Hiroshima, Japan

**Keywords:** Feeding, fermentation, rice bran, rumen liquor, phytate-P

## Abstract

**Objective:**

The objectives of this research are to overcome the limitations of rice bran (RB) and de-oiled rice bran (DORB) by fermentation anaerobically using inoculum from the rumen of a canulated sheep for desirable chemical changes.

**Materials and Methods:**

Initially, RB and DORB were fermented by 10% rumen liquor for 12 h at 39°C at different moisture levels (10, 20, 30, 40, 50, and 60% phosphate buffer). Again, DORB was fermented for 24, 48, and 72 h at 39°C using 10% rumen liquor at different moisture levels (10, 20, 30, 40, 50, and 60% phosphate buffer). Before and after fermentation, RB and DORB were analyzed for pH, proximate components, neutral detergent fiber (NDF), total-P, inorganic-P, and phytate-P.

**Results:**

Fermentation of RB and DORB for 12 h reduced (*p* < 0.05) pH, crude fiber (CF),NDF, and phytate-P, but increased (*p* < 0.05) the content of inorganic-P. Subsequent fermentation of DORB for 24, 48, and 72 h reduced pH, CF, and NDF. Total-P of fermented DORB remained similar till 72 h fermentation (*p* < 0.05). But, inorganic-P increased with the increasing duration (24, 48, and 72 h) of fermentation and increased (30, 40, and 50) moisture level (*p* < 0.05). Alternatively, phytate-P decreased with increasing duration and moisture level (*p* < 0.05).

**Conclusion:**

Inoculation of rumen microbes and incubation of RB (12 h) and DORB (24 h) at room temperature reduced phytate-P and fiber content (CF and NDF) when the moisture level was up to 50%; those are the indicators to reduce the limitation of RB and DORB to use as feed for non-ruminant animals like poultry and pigs.

## Introduction

Annual paddy rice (*Oryza sativa*) production in the world is more than 600 million metric tons, and out of this enormous amount, rice bran (RB), one of its primary by-products, accounts for about 48 million metric tons [1,2]. Globally, 63–76 million tons of RB are produced, and more than 90% is sold cheaply as animal feed [3]. RB is the major by-product in the rice milling process, containing a variety of nutrients, including moisture (10–15%), protein (14–16%), dietary fiber (25–40%), oil (15–20%), oligosaccharides (6.5%), other carbohydrates (35–55%), silica (7–10%), phenolic compounds (9.60–81.85 mg GAE/gm), and other micro-elements [4–6]. Sometimes it is considered unsuitable for human consumption but is largely used as a supplement for ruminant feeds due to its high fiber content, possible hull contamination, and susceptibility to rancidity if kept for a long time [7]. To increase the utilization of RB as poultry feed, different techniques have been established, such as fermentation [8], enzyme supplementation [9], and the inclusion of fermented products. Fermentation is one of the most promising techniques to decrease the fiber content of RB and de-oiled rice bran (DORB) [7]. Fermentation of RB increases dry matter (DM), ash, and ether extract(EE) content and reduces crude fiber (CF) content from 12.99% to 10.68% [10,11]. Moreover, two-thirds of the phosphorus (P) present in RB is available as phytate-P [12]. It is often around 80% in many cases and unavailable in poultry and pigs due to a lack of phytase in the digestion process [13]. Therefore, a large portion of the dietary P of RB cannot be utilized and is excreted in the feces. DORB has a similar limitation because it’s a by-product of an oil mill after the extraction of oil from RB. Although both RB and DORB have limitations in the diets of non-ruminants and poultry, these limitations may be overcome by fermentation by giving ruminal inoculation, as suggested by some researchers [14].

Ruminal inoculate contains 40–60% of total microbial biomass in the rumen from bacteria and protozoa and produces various fiber degradation enzymes such as -amylase, galactosidase, hemicellulases, cellulase, and xylanase [15]. Phytase enzymes are produced in the fermentation medium, which may reduce the phytate phosphorus and raise the available phosphorus content [16]. Therefore, unavailable P in RB and DORB would be converted to the available state after fermentation by rumen inoculates. Considering the positive effect of rumen bacteria on changing the chemical composition, both RB and DORB were fermented using rumen inoculums at different moisture levels and durations at 39.0°C. The chemical composition [CF, neutral detergent fiber (NDF), phytate-P, inorganic-P, and organic-P] of RB and DORB were determined before and after fermentation to know the desirable changes in nutritive value.

## Materials and Methods

### Ethical approval

Hiroshima University has its own cannulated sheep as a source of ruminal inoculum. Otherwise, no animal is involved, but the study involved fermentation in the laboratory and some chemical analysis.

### Chemicals

Most of the chemicals were provided by Wako Pure Chemical Industries Limited, Nakarai Chemicals Limited, Santoku Chemicals Industries Company Limited, and Katayama Chemicals, Japan. Firstly, RB and DORB were fermented for 12 h. Secondly, DORBs were fermented for 24, 48, and 72 h, respectively.

### Anaerobic fermentation of RB and DORB for 12 h

Full-fat RB and DORB were collected from the local market in Hiroshima, Japan. A 24-month-old male canulated Suffolk sheep (64 kg live body weight) was fed a basal diet consisting of 0.70% chopped Italian ryegrass and 0.30% concentrate on a DM basis. The amount of the basal diet was calculated to provide 1.4 times the maintenance energy requirement of the sheep [17] or 1.3 kg of DM per day. Using a suction pump, the liquid phase of the rumen was collected from the sheep, and the fluid was kept in a beaker where continuous CO_2_ was flowing to maintain the anaerobic condition and was kept in a water bath at 39°C. A buffer solution was prepared (9.8 gm NaHCO_3_, 0.04 gm CaCO_3_, 0.47 gm NaCl, 0.57 gm KCl, 3.3 gm Na_2_HPO_4_, 0.12 gm MgSO_4_.7H_2_O in 1 l distilled water) and also kept in water bath giving CO_2_ flow. Rumen fluid was filtered using cheesecloth and the filtrate was considered to innoculate diluted by the buffer at a 1:1 ratio. Then RB and DORB were mixed with water (39°C) to make 10, 20, 30, 40, 50, and 60% moisture levels where commonly 10% innoculate was mixed. A 100 gm of ready RB and DORB has fermented for 12 h anaerobically in a 200 ml plastic container in an incubator at 39°C. Then immediately transferred to the refrigerator to stop further fermentation. The acidity of fermented RB and DORB was determined using a pH meter (CyberScan 6500, Thermo Scientific, Japan). Proximate components [crude protein (CP), CF, EE, nitrogen-free extract (NFE), ash, NDF[18], total-P, inorganic-P, and phytate-P] were determined [19]. 

### Anaerobic fermentation of DORB for 24, 48, and 72 h

DORB was fermented as per the previous study, considering similar moisture levels (10, 20, 30, 40, 50, and 60%), but the duration was 24, 48, and 72 h. Fermentation was conducted anaerobically in a plastic container and incubated at 39°C. The fermented DORB was immediately transferred to the refrigerator at each interval to stop further fermentation until it dried. After measuring the pH of fermented DORB, the sample was transferred to an oven at 60°C for drying. CF, NDF, total-P, inorganic-P, and phytate-P of fermented DORB obtained from different duration and moisture were determined following recognized methods [18]. 

### Data analysis

All the data were analyzed for ANOVA [20] using the computer programs Excel and Statistical Package for the Social Sciences. Duncan’s new multiple range test (DMRT) was done to compare different mean values of parameters, considering significant differences at a 5% significance level (*p* < 0.05).

## Results

### Nutritive value of RB and DORB

The DM, CP, CF, EE, ash, NFE, NDF total-P, inorganic-P, and phytate-P were found to be 89.30, 16.85, 12.05, 23.32, 10.20, 37.58, 31.02, 1.98, 0.09, and 1.54%, respectively, for RB, as well as 88.73, 21.21, 12.81, 1.24, 11.76, 52.98, 44.91, 2.53, 0.11, respectively, for DORB. Phytate-P constitutes a major part of P in both RB and DORB. Most of the components in DORB were higher than in RB, with the exception of EE, which was extracted from RB in the oil industry. 

### Anaerobic fermentation of RB and DORB for 12 h

**Figure 1.**
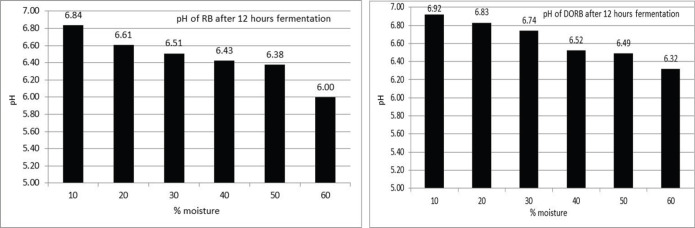
Changes of pH after 12 h anaerobic fermentation of RB and DORB (*n =* 5).

After 12 h, RB fermentation reduced the pH from 6.84 to 6.00 while increasing the moisture level from 10% to 60%. In the case of DORB, which increased from 6.92 to 6.32 as moisture levels increased from 10% to 60%, So, decreasing the pH is related to increased moisture levels for both RB and DORB examined up to 60% in this experiment (Fig. 1).

CF content decreased for every moisture level for RB and, in some cases, for DORB, as shown in Table 1 (*p* < 0.05). NDF content of RB and DORB decreased due to 12 h fermentation for every moisture level from 10% to 60% (*p* < 0.05). Overall, microbial inoculation reduces the fiber (CF and NDF) content of RB and DORB at any moisture level after 12 h of anaerobic fermentation at 39°C.

After fermentation, 30, 40, 50, and 60% of the moisture group smelled acidic for both the RB and DORB groups. Among those, 30, 40, and 50% groups were selected to analyze for total-P, inorganic-P, and phytate-P whose results are shown in Table 2. Total-P seems similar before and after fermentation at each level of moisture as well as when considering different durations for RB and DORB. Inorganic-P increased at 40% and 50% moisture levels for RB and DORB (p < 0.05). Moreover, phytate-P decreased in RB and DORB at 40% and 50% moisture levels (p < 0.05).

### Anaerobic fermentation of DORB for 24, 48 and 72 h

**Figure 2.**
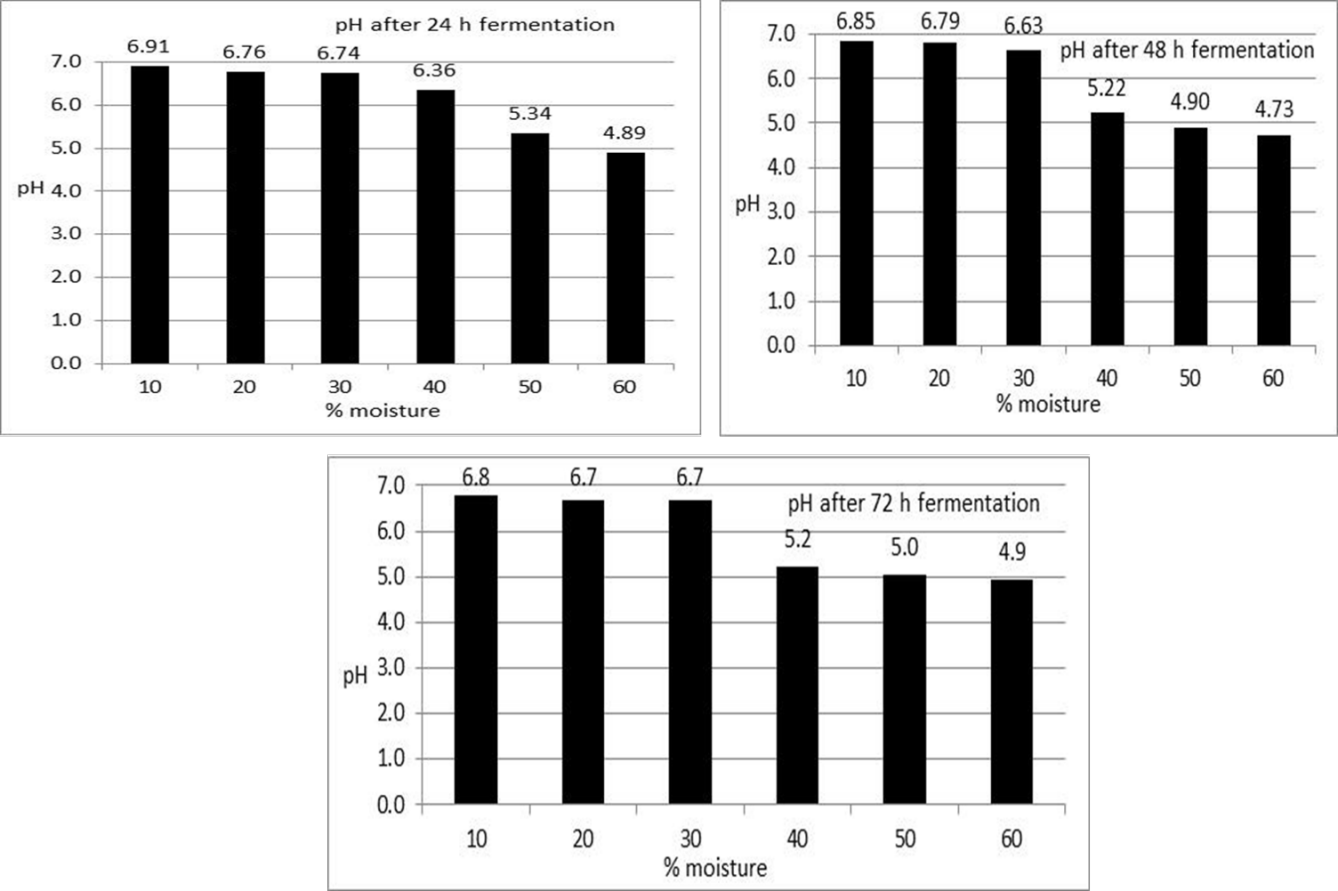
pH of fermented DORB after 24, 48, and 72 h fermentation at a different moisture level.

It was found that after 12 h fermentation of DORB pH reduced to 6.32 (Fig. 1), but further fermentation till 24, 48, and 72 h reduced to 4.89, 4.73, and 4.90 till the level of moisture 60% (Fig. 2). From the data, it is clear that for stable pH duration of fermentation is required for 24 h. Fermentation for more than 24 h did not reduce the pH of the fermented DORB. The moisture level of 60% has an effect on the reduction of pH, which is also lower within 24 h of fermentation. 

CF content decreased significantly in all the fermented groups at different moisture levels when considered at 24 or 72 h (Table 3). NDF content also decreased in the fermented groups at different moisture levels and for a shorter duration (*p* < 0.05).

**Table 1.** Changes of CF and NDF after 12 h anaerobic fermentation of RB with the different moisture levels.

Moisture (%)	0	10	20	30	40	50	60
Crude fiber							
RB	12.02^a ^± 1.04	10.06^b ^± 0.1	10.41^b ^± 0.6	10.48^b ^± 0.2	10.55^b ^± 0.2	10.41^b ^± 0.2	10.46^b ^± 0.3
DORB	13.06^a ^± 0.44	11.84^b ^± 0.37	11.75^b ^± 0.30	11.65^b ^± 0.78	11.7^ab ^± 0.08	10.69^c ^± 1.75	10.04^c ^± 2.23
Neutral detergent fiber							
RB	31.02^a ^± 1.04	26.78^bcd ^± 0.13	26.26^cd ^± 0.15	26.10^cd ^± 0.54	25.70^d ^± 0.89	27.70^bc ^± 0.43	28.10^b ^± 0.52
DORB	44.9^a ^± 0.44	31.04^b ^± 0.41	30.37^b ^±0.13	30.90^b ^± 1.14	31.40^b ^± 0.97	31.33^b ^± 1.03	31.31^b ^± 3.05

^a–d^ Mean values within the same row with different superscripts are significantly different (*p* < 0.05) (*n =* 5).

**Table 2.** Total-P, inorganic-P, and phytate-P of RB, and DORB before and after 12 h anaerobic fermentation with different moisture levels.

	RB (before fermentation)	Moisture (%)				
		30		40		50
		(after fermentation)				
Total-P	1.98^a^ ± 0.19	2.11^a^ ± 0.08	2.09^a ^± 0.19		2.11^a^ ± 0.10	
Inorganic-P	0.09^c ^± 0.00	0.09^c^ ± 0.01	0.16^b^ ± 0.01		0.24^a^ ± 0.01	
Phytate-P	1.54^a ^± 0.11	1.49^a^ ± 0.08	1.17^b^ ± 0.04		1.14^b^ ± 0.07	
	DORB (before fermentation)	Moisture (%)				
		30	40		50	
		(after fermentation)				
Total-P	2.53^a^ ± 0.28	2.60^a^ ± 0.09	2.60^a^ ± 0.51		2.70^a^ ± 0.02	
Inorganic-P	0.11^c^ ± 0.00	0.11^c^ ± 0.01	0.20^b^ ± 0.01		0.28^a^ ± 0.01	
Phytate-P	1.70^a^ ± 0.03	1.66^a^ ± 0.19	1.30^b^ ± 0.17		1.26^b^ ± 0.03	

^a–c^ Mean values within the same row with different superscripts are significantly different (*p* < 0.05) (*n =* 5).

Total-P of the DORB remained unchanged at different levels (30, 40, and 50%) of moisture for any duration (24, 48, and 72 h) of fermentation (Table 4). After 24 h of fermentation, inorganic-P increased at a moisture level of 40 and 50% (*p* < 0.05). After 48 h, fermentation inorganic-P increased with the increased moisture level (*p* < 0.05). There is also an increasing trend of inorganic-P at different moisture levels after 72 h fermentation. The phytate-P level decreased immediately after 24 h fermentation. For each duration (24, 48, and 72 h), there was significantly less phytate-P at the level of 50% moisture. 

**Table 3.** Changes of CF and NDF of DORB after anaerobic fermentation in different moisture levels at different duration.

Duration	Without fermentation	Moisture (%)					
		10	20	30	40	50	60
		After fermentation					
CF							
24	13.06^a ^± 0.44	10.02^b ^± 0.11	10.26^b ^± 0.13	10.21^b ^±0.11	10.27^b ^± 0.60	10.46^b ^± 0.31	10.26^b ^± 0.17
72	13.06^a ^± 0.44	11.09^b ^± 0.13	11.46^b ^± 0.04	11.50^b ^±0.66	11.39^b ^± 0.12	11.13^b ^± 0.17	11.18^b ^± 0.26
NDF							
24	44.9^a ^± 0.44	37.76^b ^± 0.43	37.96^b ^± 0.67	39.72^b ^± 0.21	38.45^b ^± 0.60	40.94^b ^± 0.38	40.80^b ^± 0.41
72	44.9^a ^± 0.44	39.26^b ^± 0.56	38.52^b ^± 0.29	37.39^b ^± 1.64	39.47^b ^± 0.53	38.45^b ^± 0.72	39.24^b ^± 1.70

^a,b ^Mean values within the same row with different superscripts are significantly different (*p* < 0.05) (*n =* 5).

**Table 4.** Comparison of total-P, inorganic-P, and phytate-P of fermented DORB in different duration and moisture level with non-fermented DORB.

		Total-*p*	Inorganic-*p*	Phytate-*p*
	DORB (non-fermented)	2.53^a^ ± 0.28	0.11^a ^± 0.00	1.70^a^ ± 0.03
24 h (Fermented)				
% Moisture	30	2.53^a^ ± 0.15	0.11^a^ ± 0.00	1.42^a^ ± 0.05
	40	2.57^a^ ± 0.14	0.14^b^ ± 0.01	1.34^a^ ± 0.08
	50	2.57^a^ ± 0.03	0.16^c^ ± 0.01	1.21^b^ ± 0.13
48 h (Fermented)				
% Moisture	30	2.50^a ^± 0.08	0.25^b^ ± 0.01	1.42^a^ ± 0.11
	40	2.59^a ^± 0.04	0.43^c^ ± 0.06	1.42^a^ ± 0.08
	50	2.48^a ^± 0.05	0.63^d^ ± 0.06	1.19^b^ ± 0.09
72 h (Fermented)				
% Moisture	30	2.45^a^ ± 0.27	0.82^b^ ± 0.08	1.26^a ^± 0.07
	40	2.44^a ^± 0.07	1.16^c^ ± 0.13	1.23^a^ ± 0.02
	50	2.35^a ^± 0.03	1.87^d^ ± 0.17	1.17^b^ ± 0.03

^a–d ^Mean values within the same column (for each duration) with different superscripts are significantly different (*p* < 0.05). (*n =* 5).

## Discussion

Anaerobic fermentation of RB and DORB by rumen bacteria will increase the number of bacteria in the substrate within the stipulated period. After feeding the substrate and the bacteria, it will be digested in the gastrointestinal tract of non-ruminant animals, similar to the digestion of microbes in the lower gut of the ruminant animal. So, fermented RB and DORB produced by rumen bacteria will suit non-ruminant animals.

Fermentation of RB and DORB anaerobically for a period of 12 h using rumen liquor at 39.0°C has reduced pH (6.0 and 6.32). In the case of DORB, the pH was further reduced to 4.89, 4.73, and 4.90 after 24, 48, and 72 h fermentation at 60% moisture level. In those cases, moisture content was also a factor in lowering the pH. It was found that a 60% moisture level and a 24 h duration were suitable for lowering the pH of RB and DORB. The pH reduction was mainly due to the production of volatile fatty acids (VFAs) and lactic acid [21,22]. Other researchers indicated that the changes in pH were due to the production of a sugar molecule in an equimolar mixture of organic acids [23,24], ethanol, and carbon dioxide by microorganisms [22] in the closed fermentation medium [25]. The VFA produced in the rumen ecosystem is used by microorganisms to synthesize body protein. Still, as the fermentation has been conducted outside the rumen, the VFAs trapped in the substrate cause a lowering of the fermented RB and DORB, which is reflected in the lowering of the pH.

CF and NDF content decreased at every moisture level and for different durations for RB and DORB, which was supported by other research findings using bacteria from the rumen (*Ruminococcus albus and Clostridium cellulovorans*) in rice straw [26]. It would be due to the increased enzyme activity of inoculated bacteria [26,27]. The previous study also found that cellulolytic ruminococci play a major role in the breakdown of plant cell wall material in the rumen [28]. Rumen microbes can produce β-glucanases, cellulases, and hemicellulases, which are required to break down cellulose, hemicelluloses, and phenolic polymers in this experiment but not in the rumen [29,30]. Fiber is degraded by a combination of ruminal bacteria, fungi, and protozoa [31].

Interestingly, all the fermented groups showed less CF and acid detergent fiber (ADF) content than the original, which is supported by many researchers [32–34]. Most of the time, the amount of CF and ADF in fermented groups stays the same, even when the moisture level or time of fermentation changes. Still, in the case of ruminal fermentation, it should be reduced. It is debatable whether to reduce further despite the increased duration and level of moisture. In that case, pH is one of the most important factors for ruminal microorganisms in the fermentation of fibrous components, and it should be between 6 and 7 [24,35]. But, after a certain period, the pH was reduced and remained stable after a certain period. Also, the pH of the rumen depends on several factors, e.g., the production of saliva, the absorption of VFAs, the level of feed intake, and the exchange of buffer through the rumen wall [35]. In the used *in-vitro* system, most likely the pH was reduced due to the formation of VFA [24,36,37] and lactic acids [24,36] during fermentation and their accumulation [23]. So, further reductions of CF and NDF were not observed due to the inhibition of cellulolytic bacteria. As poultry cannot break down cellulose [38], fermented RB with reduced fiber could be a useful ingredient in poultry feed.

Increased inorganic content would be related to the decreasing phytate-P after the fermentation of RB and DORB. Therefore, fermentation at 39°C at 60% moisture level improved nutritive value when fermented up to 72 h. Fiber and phytate-P levels were decreased in all the fermented groups compared to the control group (*p* < 0.05). This finding is consistent with previous findings that the fermentation of RB with rumen liquor can reduce phytate phosphorus content [32]. Another study also reported that the fermentation of RB using rumen liquor could improve phosphorus concentration by about 7.5% [8]. Phytate-P content of DORB was 1.39% found in a study, which was similar to the content of this finding [39]. Some researchers found that the phytate–degrading enzymes from RB were active in the first 6 h of the process [40]. So, rumen inoculation and fermentation reduced the phytate-P content of RB and DORB, which is also found in this study.

## Conclusion

Anaerobic fermentation of RB at 12 h using rumen liquor reduced the fiber and phytate-P content at a 50% moisture level. In contrast, DORB fermented for 24 h with rumen liquor showed greater inorganic-P at the same moisture level and reduced pH, fiber, and phytate-P; these are the indicators to minimize the limitations of RB and DORB for use as feed for non-ruminant animals like poultry and pigs. Further research is necessary to study the effects of specific microbes and changes in single-cell protein and bioavailability in non-ruminant animals. 

## List of abbreviations

RB, rice bran; DORB, de-oiled rice bran; CP, crude protein; CF, crude fiber; NFE, nitrogen-free extract; NDF, neutral detergent fiber; ADF, acid detergent fiber; DM, dry matter; EE, ether extract; VFAs, volatile fatty acids; P, phosphorus.
